# The Effect of the Ala16Val Mutation on the Secondary Structure of the Manganese Superoxide Dismutase Mitochondrial Targeting Sequence

**DOI:** 10.3390/antiox11122348

**Published:** 2022-11-27

**Authors:** Matic Broz, Veronika Furlan, Samo Lešnik, Marko Jukič, Urban Bren

**Affiliations:** 1 Faculty of Chemistry and Chemical Engineering, University of Maribor, Smetanova ulica 17, SI-2000 Maribor, Slovenia; 2 Institute of Environmental Protection and Sensors, Beloruska ulica 7, SI-2000 Maribor, Slovenia; 3 Faculty of Mathematics, Natural Sciences and Information Technologies, University of Primorska, Glagoljaška ulica 8, SI-6000 Koper, Slovenia

**Keywords:** manganese superoxide dismutase, polymorphism rs4880, mutation Ala16Val, molecular dynamics simulations, oxidative stress

## Abstract

Manganese Superoxide Dismutase (MnSOD) represents a mitochondrial protein that scavenges reactive oxygen species (ROS) responsible for oxidative stress. A known single nucleotide polymorphism (SNP) rs4880 on the SOD2 gene, causing a mutation from alanine to valine (Ala16Val) in the primary structure of immature MnSOD, has been associated with several types of cancer and other autoimmune diseases. However, no conclusive correlation has been established yet. This study aims to determine the effect of the alanine to valine mutation on the secondary structure of the MnSOD mitochondrial targeting sequence (MTS). A model for each variant of the MTS was prepared and extensively simulated with molecular dynamics simulations using the CHARMM36m force field. The results indicate that the alanine variant of the MTS preserves a uniform α-helical secondary structure favorable for the protein transport into mitochondria, whereas the valine variant quickly breaks down its α-helix. Thus, the alanine MTS represents the more active MnSOD variant, the benefits of which have yet to be determined experimentally.

## 1. Introduction

Reactive oxygen species (ROS) represent one of the mainly harmful by-products of cellular metabolism, but are also vital for proper cell functioning as activators of certain signaling pathways [1]. For instance, the stimulation of non-phagocytic cells with ligands increases intracellular ROS levels [2], the activation of peptide growth factors via tyrosine kinase [3,4], and the cytokine pathways [5,6]. While the actual targets of ROS remain largely unexplored, it has been demonstrated that non-lethal concentrations of H_2_O_2_ oxidize and thus chemically modify several proteins, including p53, Jun, Fos, and the NF-κB p50 subunit [7], thereby stimulating (p50) or inhibiting (p53, Jun, Fos) the transcriptional activity.

Antioxidants maintain a balance between ROS production and consumption by converting them to harmless reduced species; however, when this balance is disrupted, the elevated ROS levels result in increased DNA, protein, and lipid damage [7,8,9,10]. This condition, dubbed “oxidative stress”, has been linked to a variety of complex diseases, including various types of cancer [11,12,13,14], asthma [15], Crohn’s disease [16], and multiple sclerosis [7]. The primary source of cellular ROS represents the leak of electrons from the respiratory or electron transport chain (ETC) located in the mitochondria [17,18,19,20].

The human genome encodes multiple superoxide dismutase (SOD) enzymes that catalyze the transformation of superoxide anions into hydrogen peroxide [21] to counteract the harmful effects of excessive superoxide anion production. The first line of defense against respiratory chain electron leak forms the manganese superoxide dismutase (MnSOD), located in the mitochondrial matrix, where it scavenges the superoxide anions [22].

**Figure 1:**
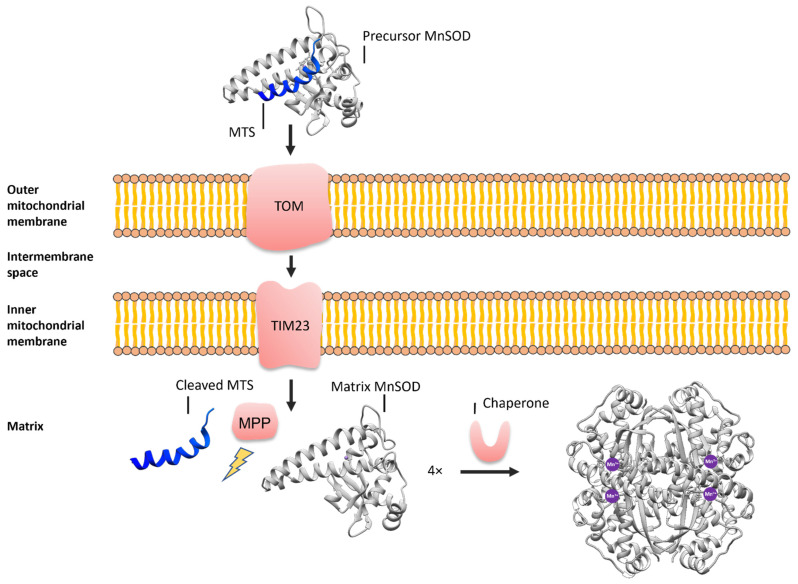
A schematic representation of the mitochondrial protein import. The precursor MnSOD reaches the outer mitochondrial membrane, where its MTS interacts with the TOM complex, which requires an amphiphilic α-helix. The TIM23 complex then transports the precursor MnSOD across the inner mitochondrial membrane into the mitochondrial matrix, where the MPP cleaves off the MTS. Chaperones then fold MnSOD into its active form and insert one Mn^3+^ ion per monomer.

The human MnSOD is encoded by the SOD2 gene located on the sixth chromosome in the region 6q25.3 [23]. In its mature state, the human MnSOD forms a tetrameric enzyme comprised of four identical subunits, each consisting of 198 amino acids and one Mn^3+^ ion [24]. The mitochondrial import machinery is required to transport proteins destined for the mitochondria (e.g., MnSOD) across two membranes, the outer and inner mitochondrial membranes [25]. For proteins to cross the membrane, specific amino acid sequences called mitochondrial targeting sequences (MTSs) are required, which fall into two categories: the N-terminal and internal targeting sequences [26]. MTSs have been shown to form amphipathic α-helices, which are then recognized by mitochondrial translocases of the outer membrane (TOM) (Figure 1) [27,28,29]. Upon maturation in mitochondria, the mitochondrial proteins containing N-terminal sequences (e.g., MnSOD) are cleaved of their MTS by the mitochondrial processing peptidase (MPP). It has also been suggested that MTSs adopt context-dependent conformations—α-helical conformation for the recognition by the mitochondrial import machinery, and an extended conformation for the cleavage [30]. Finally, chaperones fold these proteins into their active form and insert metallocofactors [31].

**Figure 2:**
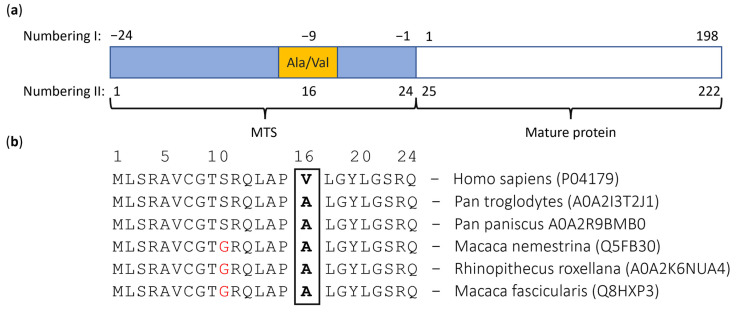
A schematic representation of the different systems of numbering and the conservation of the MTS sequence in homo sapiens and other primate species. (**a**) MTS (blue), Ala16Val (yellow), and mature MnSOD (white). The protein residues can be numbered with respect to the initial residue of the mature protein (numbering I) or with respect to the initial residue of the MTS of the immature protein (numbering II). Consequently, the Ala-9Val (numbering I) is equivalent to the Ala16Val (numbering II). (**b**) The MTS sequence shows high interspecies conservation. Red letters represent the amino acids that differ from the human MTS of MnSOD, while the bolded letter represents the location of the Ala16Val substitution. In all other primate species, only alanine can be found in position 16; the same trend can also be observed for most more distant species (Figure S3J).

In 1996, a mutation from cytosine (C) to thymine (T) in the coding region of the SOD2 gene was discovered by two independent studies: Shimoda-Matsubayashi, S. et al. [32], in the Japanese population, and Rosenblum, J.S. et al. [33]. This mutation at position 47 of the SOD2 gene results in a substitution of alanine with valine (Ala16Val, also dubbed Ala-9Val) at position −9 of the protein, which corresponds to position 16 in the MTS of MnSOD, as shown in Figure 2a. In this study, we adhere to numbering II.

Figure 2b depicts the comparison between the MTSs of different primates. Among primates, only the substitution located at position 11 from serine to glycine (S → G) is observed, indicating an otherwise high level of interspecies similarity (for human, the V variant is used in sequence comparisons as this is the canonical sequence deposited as UniProt entry P04179). There are no Uniprot entries containing valine at position 16 among primates other than Homo sapiens. Expanding to other organisms shown in Figure S3, we observed that only three of the twenty-four examined Uniprot entries included valine at position 16 (12.5%) rather than alanine (87.5%); no other amino acid has been observed at position 16. Moreover, we found that the two adjacent amino acid residues (Pro15 and Leu17) are highly conserved across all examined species (Supplementary material Figure S3).

The MTS is required for the MnSOD to be correctly localized in the mitochondrial matrix. Upon successful localization, the MTS is cleaved off the mature MnSOD and decays, making it a highly unstable species with an unresolved structure. As a result, the human MnSOD possesses 34 inputs in the online RCSB protein data bank, but only two represent MnSOD in its native state (PDB ID: 2ADQ, 5VF9). Despite a large number of entries, the structure of MnSOD’s MTS has not been determined due to the short-lived nature of the MnSOD precursor. Therefore, we performed molecular dynamics (MD) simulations to reveal the nature of the MTS secondary structures.

Shimoda-Matsubayashi, S. et al. [32] proposed a typical amphiphilic helical structure of the Ala-MTS and its interruption in the Val-MTS variant using the Chou-Fasman method [34] and the helical-wheel method [35]. Moreover, an analysis of 23 MTSs revealed that amphiphilic helices in the MTS may indeed be required for the import of immature MnSOD into mitochondria [29]. Therefore, the alanine variant of MnSOD should be preferred for mitochondrial transport.

However, there is a disagreement about the transport properties of the two immature MnSOD variants. On the one hand, Sutton, A. et al. showed that the Val-MnSOD precursor might be partially arrested in the inner mitochondrial membrane (IMM) and produces 30–40% less active MnSOD than the Ala-MnSOD precursor [36]. A follow-up study indeed demonstrated four-fold higher levels of the mature exogenous protein of the Ala variant compared to the Val variant in transfected HuH7 human hepatoma cells [37]. Conversely, Bastakia, M. et al. discovered that the TT (thymine-thymine, translating to valine-valine) and CT (cytosine-thymine, translating to alanine-valine) genotypes of the polymorphic MnSOD rs4880 confer a higher enzymatic activity in human erythrocytes than the CC (cytosine-cytosine, translating to alanine-alanine) genotype [38].

Single nucleotide polymorphism (SNP) rs4880 has been associated with various complex diseases. For instance, the Val allele has been associated with a 10-fold increased risk of heart disease in patients with hereditary hemochromatosis [39], a 2.3-fold increased risk of developing aggressive forms of prostate cancer in patients consuming more iron [40], and an increased risk of lung cancer in the Turkish population [41]. On the other hand, the Ala/Ala genotype has been associated with an increased risk of malignant pleural mesothelioma (MPM) [42]. In addition, the carriers of the Ala allele have been found to have an increased risk of developing acoustic neuroma [43], a lower survival rate when treated with cyclophosphamide for breast cancer [44], an increased risk of intestinal metaplasia [45], a shorter median overall survival after postoperative chemotherapy with platinum and fluorouracil against gastric cancer [46], increased ROS-induced DNA damage biomarkers [47], increased oxidative damage from polyaromatic hydrocarbon (PAH) exposure [48], and a 4-fold increased risk of breast cancer in premenopausal women [49]. The rs4880 SNP and its associations with diseases are summarized in Table 1.

Both variants are present natively in the human population (SOD2 gene) according to SNPedia [50,51,52]. Based on the results of our study (Figure 2b), we adhere to the Ala16Val notation throughout this paper for the reason of clarity.

Since the experimental structural data on the MnSOD MTS is currently unavailable, we investigated the effect of the Ala16Val SNP on the secondary structure of the MnSOD MTS via extensive MD simulations. We obtained the initial MTS secondary structural information with PsiPred (v4.0) [53], as well as by using an online server for protein structure prediction (PEP-FOLD3) [54]. The resulting MTS 3D structures were then docked to the native MnSOD structure (PDB ID: 5VF9) [55] using Hdock (13 November 2021) [56] to form the initial MnSOD MTS structure. The resulting structures were solvated, equilibrated, and propagated in time using 200 ns MD simulations (CHARMM36m) [57]. The ultimate goal was to evaluate the secondary structure dynamics of the Val variant and compare it to the native Ala variant.

## 2. Materials and Methods

The effect of the Ala16Val substitution on the secondary structure of the MnSOD MTS was followed using extensive MD simulations using the CHARMM36m [57] force field in NAMD [58]. At the time of writing this article, the RCSB database [59] contained 34 human MnSOD structures resolved by X-ray or neutron diffraction. However, all structures were based on the mature MnSOD protein, missing the MTS. Therefore, the structure of the MTS has not yet been elucidated by either X-ray crystallography or nuclear magnetic resonance (NMR). For this reason, the highest resolution RCSB entry of the native human MnSOD (PDB ID: 5VF9) [55] was applied in this study.

### 2.1. Force Field Comparison

Before we executed MD simulations on the whole protein, a comparison between Amber [60], CHARMM, and GROMOS [61] force fields was performed with 200 ns MD simulations of the MTS only. The inputs for MD simulations were prepared in the same fashion as described in Section 2.4. for the whole protein. After 200 ns, the obtained MD trajectories were analyzed. The CHARMM force field was selected for the subsequent simulation due to the stability of its MD trajectories (Figure S1a) and the preservation of the secondary structures (Figures S1b and S2).

### 2.2. MTS Construction

The initial MTS structure was obtained using the online de novo peptide structure prediction server PEP-FOLD3, version 3.5 [54]. FASTA sequence of the MTS served as input, and 1000 simulations were carried out for each MTS variant. For each variant, models were sorted according to their sOPEP score, and the highest-scoring uniform α-helical structure was applied in subsequent docking and MD experiments.

### 2.3. Docking of the MTS to the MnSOD

**Figure 3:**
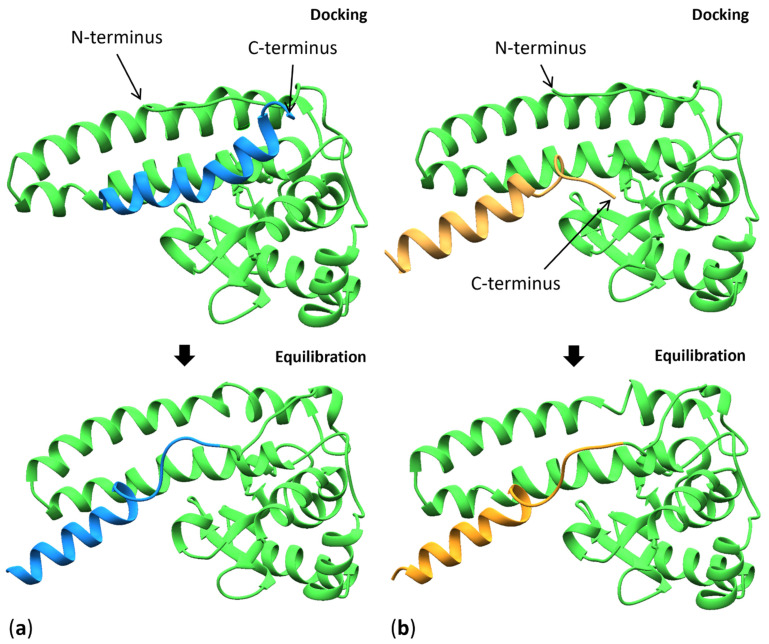
A cartoon representation of the docking and post-minimization results used in MD for (**a**) the alanine and (**b**) the valine variants. The C terminus of the MTS (blue and orange) and the protein’s N terminus (green) are annotated.

Because the relative pose of MnSOD’s MTS is unknown, the MTS variant models generated by PEP-FOLD3 were docked into the known protein structure (PDB ID: 5VF9) using HDOCK (version 2021-11-13) [56]. HDOCK performs geometric matching between the receptor and ligand-protein surfaces represented by coarse-grained models with subsequent knowledge-based scoring by the ITScorePP function [62,63]. The MnSOD protein (residues 25–222) served as the input receptor molecule, and the PEP-FOLD3 MTS model (residues 1–24) served as the input ligand molecule for each variant. The models with the highest scores for each variant exhibiting a plausible MTS C-terminus location (close to the MnSOD N-terminus), allowing for the loop modeling in the subsequent step, were selected (Figure 3). The connecting loops (23–31) were modeled using the Modeller software (10.2) plugin in the UCSF Chimera [64,65]. Both MTS structures equilibrated using MD simulations to a similar pose as the one seen in the initial Ala-MTS docked structure, indicating the validity of the initial structure guesses.

### 2.4. Molecular Dynamics Simulations

The CHARMM Gui was used to generate the starting coordinates for both variants as well as input files for the CHARMM36m force field in the NAMD. In our MD simulations, we modeled the manganese ion using CHARMM Small Molecule Library’s (CSML) MN3P parameters on the basis of Azadmanesh, J. et al. [55], and Borgstahl, G.E.O et al. [66]. Namely, the metal cofactor is oxidized or reduced by a ligand bound directly to the metal where superoxide anion binds to the available coordination site of the manganese metal cofactor, and studies observed that MnSODs typically rest in the Mn^3+^ oxidation state according to Barnese, K. et al. [67] and Stroupe, M.E. et al. [68].

The proteins were solvated in a rectangular water box with 90.0 Å sides and neutralized in 0.15 M NaCl using the Monte Carlo placing method. Additionally, WYF parameters for cation-pi interactions were included [69]. Both variants were first minimized using 50 steps of steepest descent (SD) and Adopted Basis Newton-Raphson (ABNR), followed by heating using the Nosé-Hoover method [70], and a 125 ps equilibration at constant volume using the Velocity Verlet algorithm (VVER). The equilibration of the systems was accomplished by applying an NVT ensemble at 310.15 K and the integration time-step of 1 ns. The production was carried out with the Leapfrog Verlet integration for 200 ns using an NPT ensemble, with the Hoover thermostat set to 310.15 K, the time step to 2 fs, and a periodic boundary condition imposed along with the particle mesh Ewald method [71] to treat long-range electrostatics. The mass of the thermal piston for the Hoover thermostat was set to 2000.0 ($\frac{\mathrm{kcal}}{\mathrm{mol}}ps^{2}$), while the Langevin piston bath temperature and the initial temperature at which the velocities have to be assigned to begin the dynamics run were set to 310.15 K. The barostat was set to 1.0 atmosphere, the mass of the pressure piston was defined as the total of selected atoms divided by 50.0, and the Langevin piston collision frequency was set to 20.0. A miscellaneous mean-field potential (MMFP) was applied to all protein non-hydrogen and non-ion atoms using a spherical harmonic potential, while the covalent bonds to hydrogens were constrained using the SHAKE algorithm.

## 3. Results and Discussion

**Figure 4:**
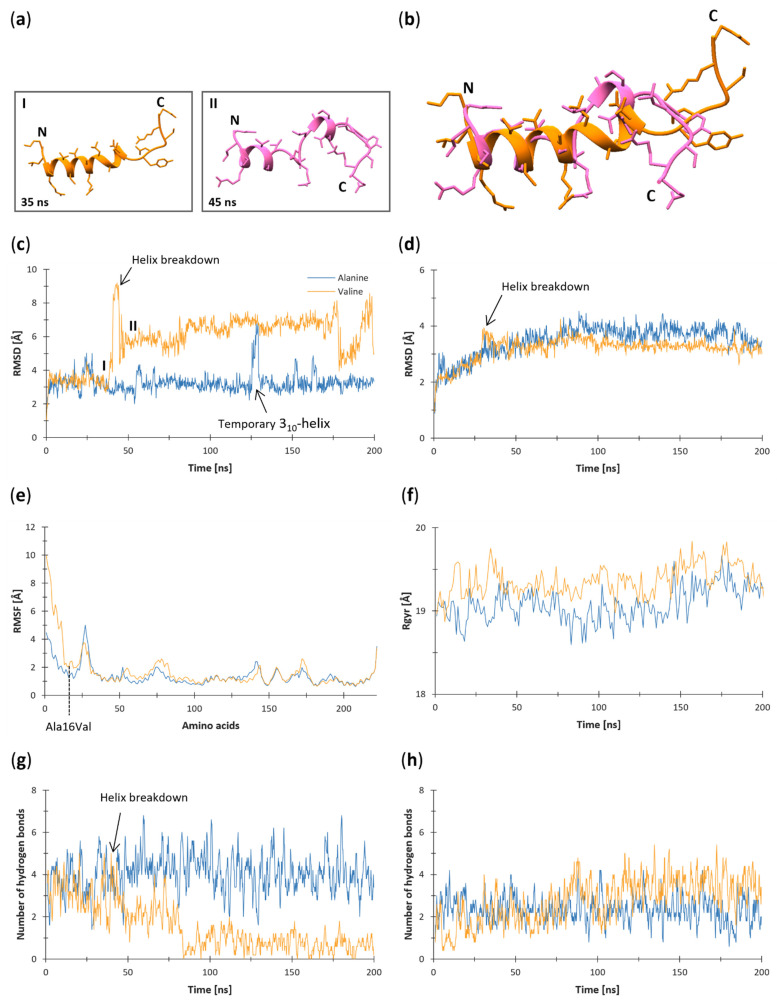
Cartoon representation of the Val-MTS before (35 ns) and after (45 ns) the helix breakdown and graphical representation of RMSD, RMSF, Rgyr, and hydrogen bond analysis during the 200 ns MD simulations. (**a**) Cartoons depict the MTS helix right before and after the breakdown and (**b**) their aligned comparison. Graphs depict the data for (**c**) RMSD of the MTS sequences (amino acid residues 1–24), (**d**) RMSD of the rest of the protein (amino acid residues 25–222), (**e**) RMSF based on the position of the alpha carbon, and for (**f**) the radius of gyration. (**g**) The number of intramolecular (between the MTS residues) and (**h**) intermolecular (between the MTS and the rest of the protein) hydrogen bonds are represented using a moving average with a period of five due to high fluctuations. The alanine variant is denoted in blue, while the valine variant is in orange color.

The root-mean-square deviation (RMSD) (Figure 4c,d), the root-mean-square fluctuation (RMSF) (Figure 4e), the radius of gyration (Rgyr) (Figure 4f), the number of hydrogen bonds within the MTS (residues 1–24) (Figure 4g), and with the rest of the protein (Figure 4h) during the MD trajectories were studied in VMD [72] using the STRIDE plugin [73]. Numerical data were analyzed and graphed in Microsoft Excel (Office 16) [74].

Figure 4a depicts the Val-MTS structure before (I) and after (II) the helix breakdown (Figure 4c and as shown in Supporting Information Movies S1 and S2), revealing a uniform helical structure before (I) and two divided shorter helices after (II). The aligned comparison of both structures (Figure 2b) indicates that the *N*-termini of the Val-MTS are well-aligned, whereas the C-termini point in the opposite directions due to the unfolding of the helix.

### 3.1. Root-Mean-Square Deviation (RMSD)

**Figure 5:**
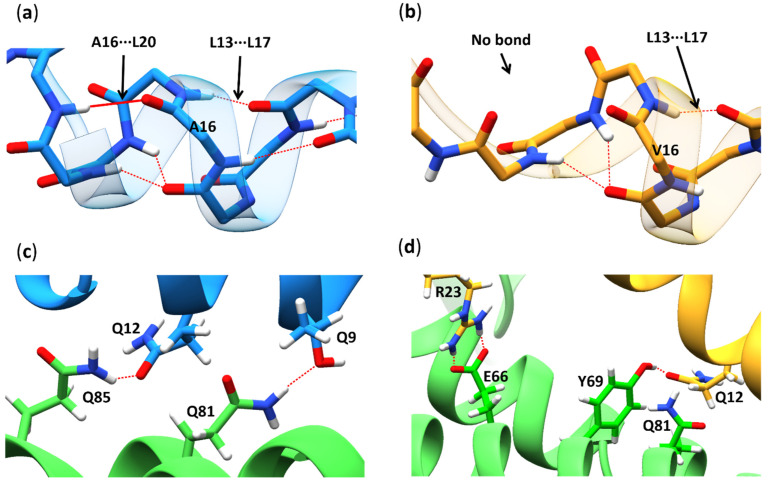
Visual representation of the essential hydrogen bonds. (**a**) Ala16 formed a standard α-helical hydrogen bond with Leu20 (solid red line) already at the beginning of the MD simulation (at 1 ns), and it remained the most stable hydrogen bond throughout the trajectory (Table 2). (**b**) Val16 did not form a hydrogen bond with Leu20 during the MD simulation. (**c**) The MTS α-helix was stabilized by forming intermolecular hydrogen bonds with the MnSOD protein α-helix (amino acid residues 78–103) Thr9···Gln81 and Gln12···Gln85 for the alanine variant, (**d**) and Gln12···Tyr69 and Gln12···Gln81 for the valine variant, but never concurrently. Both MTSs were held in place by the intermolecular Arg23···Glu66 hydrogen bonds. Dotted red lines depict hydrogen bonds, blue color the Ala-MTS, yellow color the Val-MTS, and green color the rest of the MnSOD protein.

**Figure 6:**
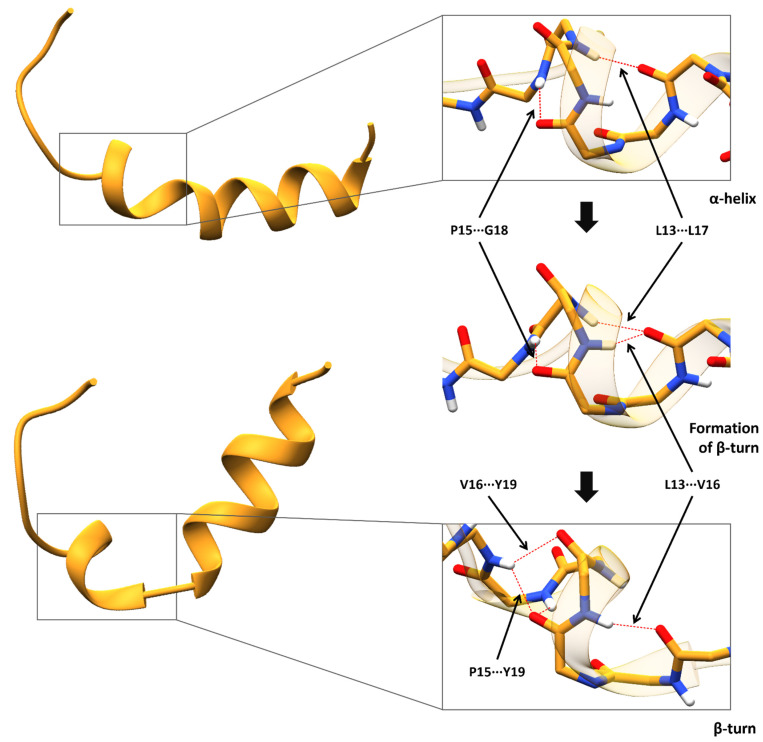
Schematic representation of the hydrogen bonds responsible for the Val-MTS helix breakdown. The collapse of the hydrogen bond between Leu13 and Leu17 seems to be responsible for the conversion of Val-MTS to a β-turn at residue 13, which initiated the breakdown of the α-helix. Β-turn is defined as an i, i + 3 segment possessing a hydrogen bond between the CO group of residue i and the NH group of residue i + 3.

In our study, the root-mean-square deviation (RMSD) was calculated as the difference between the backbone atoms of the models’ initial structural conformations and those generated by MD simulations using the VMD’s RMSD trajectory tool. The smaller the deviations, the more stable the protein structures. From the RMSD values obtained for both MTSs (amino acid residues 1–24; Figure 4c), it can be seen that both systems initially reached an equilibrium at around 1 ns with an RMSD value of 3.8 Å. The Ala-MTS remained stable throughout the 200 ns MD simulation because it maintained the Ala16···Leu20 hydrogen bond (Figure 5a, Table 2). However, a significant increase in the RMSD by around 3.2 Å can be observed when the α-helix transiently broke into a 3_10_-helix. After this, at the 125th ns, the Ala-MTS α-helix restored itself and the system re-equilibrated at 3.8 Å, indicating that the alanine variant exhibits a high affinity for the α-helix. On the contrary, the Val-MTS underwent an abrupt increase in the RMSD values of around 5.3 Å at 40 ns since the Val16···Leu20 hydrogen bond was not formed (Figure 5b, Table 2). At this point, the Val-MTS adopted a U-shape starting at residue 13, whose secondary structure changed from an α-helix to a β-turn (Figure 6). After the Val-MTS stopped bending at 50 ns, its RMSD value dropped to around 3.0 Å above the initial equilibrium state but continued to increase throughout the MD simulation as the α-helix collapsed further, indicating an unstable structure.

Based on the RMSD values calculated for the rest of the protein (amino acid residues 25–222; Figure 4d), it can be seen that the system reached an equilibrium after 85 ns for both variants and maintained at around 3.2 Å until the end of the MD simulations. However, a transient increase in RMSD of around 1 Å can be observed for the MnSOD protein of the valine variant caused by the Val-MTS α-helix breakdown (Figure 4a,b,d). As a result, the protein might contribute to the stability of the MTS. However, the helix degradation of the MTS has, as expected, no long-term effects on the rest of the MnSOD protein.

### 3.2. Root-Mean-Square Fluctuation (RMSF)

Root-mean-square fluctuation (RMSF) was measured for each C_α_ atom of the MnSOD backbone as the difference between its initial position and the positions generated during the MD simulations. It was calculated using the “measure” command of VMD. The RMSF values of protein amino acid residues 25–222 were very similar for the alanine and valine models (Figure 4e). However, as expected from the high RMSD values (Figure 4c), the RMSF values of the MTS amino acid residues 1–24 varied considerably. Interestingly, the RMSF plot approached a local minimum at the crucial residue 16, suggesting that it may have little effect on the MTS’s secondary structure but instead controls the secondary structures of the neighboring residues through the formation of hydrogen bonds. Moreover, the RMSF curve of both models peaks at residues 25–30, indicating the presence of a loop. The average RMSF values of the MTS indicate that the alanine variant (2.26 Å) is much more stable than the valine variant (4.41 Å), while the rest of the protein is most likely not affected by the Ala16Val mutation. The high RMSF values of the Val-MTS can be attributed to the helix breakdown at 40 ns, thus allowing the amino acid residues 1–12 to move more freely, which can also be observed in the graphical representation of the RMSF (Figure 4e).

### 3.3. Radius of Gyration (Rgyr)

The radius of gyration (Rgyr) was calculated in VMD using a custom script. Rgyr indicates how tightly packed a given protein is, pointing to its stability. From the Rgyr values calculated for both protein variants (Figure 4f), both models exhibited a gradual increase in Rgyr values throughout the MD simulations with high fluctuations. However, the alanine model displayed a lower average Rgyr value (19.10 Å) than the valine model (19.32 Å). Notably, both models entered the MD simulation with nearly identical Rgyr values (18.94 Å for the alanine; 18.96 Å for the valine variant) but quickly diverged as the Rgyr values for the valine model increased, while the alanine model’s Rgyr values decreased. Despite a significant deviation in Rgyr values during the MD simulations, they basically converged at the end of the 200 ns trajectory. The more compact protein structure of the alanine variant throughout the MD simulation signifies its greater stability compared to the valine variant.

### 3.4. Hydrogen Bonds

The hydrogen bonds of both variants were analyzed using the Hydrogen Bonds plugin of VMD. The default parameters (D-A distance 3.0 Å, D-H-A angle 20.0°) were used for the analysis. Hydrogen bonds were sorted by their occupancy (%), as shown in Table 2. On average, the Ala-MTS formed significantly more intramolecular hydrogen bonds (4.05) than the Val-MTS (1.55) (Figure 4g). Notably, at 40 ns, the number of intramolecular hydrogen bonds within the Val-MTS amino acid residues started to decrease, indicating the α-helix breakdown. The number of intramolecular hydrogen bonds in the Val-MTS kept decreasing until around the 80th ns, when it stabilized at less than one on average. Since the Ala-MTS contained more than 2.5 times as many intramolecular hydrogen bonds as the Val-MTS, the alanine variant remained much more stable. On the contrary, the average number of intramolecular hydrogen bonds between the MTS and the rest of the protein did not differ significantly (2.33 for the alanine, 2.80 for the valine variant) (Figure 4h). In fact, the Val-MTS formed more hydrogen bonds with the rest of the protein, which could be attributed to its less compact structure, and thus, a larger surface available for hydrogen bonding.

From the hydrogen bond occupancy analysis (Table 2), it can be observed that Ala16 formed a vital bond with Leu20 (Figure 5a) 43.5% of the time, but Val16 did not (Figure 5b). Based on this distinction, we can safely assume that the Ala16···Leu20 hydrogen bond may be crucial for the stability of the α-helix. Another important intramolecular hydrogen bond within the MTS amino acid residues formed between Leu13 and Leu17, with a lower occupancy in the Val-MTS (25.4%) than in the Ala-MTS (39.6%), but it was present in both variants from the start to the finish of MD trajectory (Figure 5a,b).

Both MTS variants formed few hydrogen bonds with the rest of the protein (Table S1), which is generally considered a stabilizing factor. The most common intermolecular hydrogen bond between both MTSs and the rest of the protein was Arg23···Glu66, which lies outside the α-helical region and represents a part of the loop connecting the MTS with the MnSOD protein (Figure 5d), thus most probably holding the MTS in place. The central stabilizing intermolecular hydrogen bonds between the helical region of the MTS and the MnSOD protein were Thr9···Gln81 (16.20%) and Gln12···Gln85 (3.60%) for the alanine variant (Figure 5c), and Gln12···Tyr69 (35.10%) or Gln12···Gln81 (7.90%) for the valine variant (Figure 5d). All protein structures are drawn in a cartoon or stick representation and rendered using UCSF ChimeraX 1.15 (build 42258) [64].

The breakdown of the α-helix was observed during the valine MD simulations as opposed to the alanine variant (Figure 5b). Leu13 does not form a stable hydrogen bond with Leu17 in the valine variant, which is characteristic of a typical α-helical secondary structure. Instead, in the MD simulations of the valine variant, a new hydrogen bond between Leu13 and Val16 gets established, resulting in the formation of a β-turn at residue 13 (Figure 6). Furthermore, the initial hydrogen bond between Pro15 and Gly18 gets replaced with a hydrogen bond between Pro15 and Tyr19. Moreover, a new hydrogen bond between Val16 and Tyr 19 is formed.

### 3.5. Nonbonded Energy

**Figure 7:**
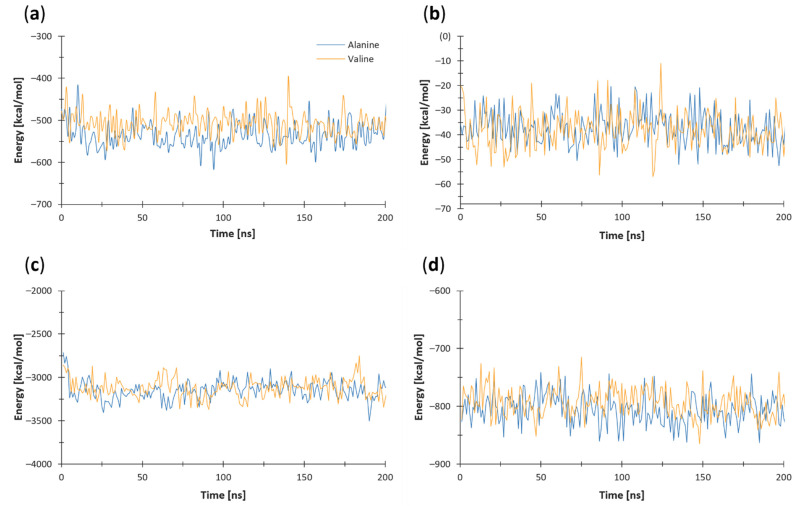
Graphical representation of the intramolecular electrostatic and vdW energy analyses. Lower values indicate a more stable structure. The (**a**) electrostatic and (**b**) van der Waals intramolecular potentials for the MTS domain. The (**c**) electrostatic and (**d**) van der Waals intramolecular potentials for the whole protein. The Ala variant is denoted in blue, while the Val variant is in orange.

The systems’ energy was calculated using the NAMD plugin for VMD, version 1.4. From the graphical representations of intramolecular nonbonded energy analyses (Figure 7), it can be observed that the Ala-MTS (−539.33 kcal/mol) is more stable than the Val-MTS (−479.20 kcal/mol) due to a lower average intramolecular electrostatic potential throughout the MD simulations (Figure 7a). The intramolecular van der Waals potential analysis did not point to a difference in stability in terms of absolute values; however, the Val-MTS exhibited higher fluctuations (Figure 7b). The total intramolecular nonbonded potential was thus significantly lower for the Ala-MTS (−577.23 kcal/mol) than for the Val-MTS (−517.25). The electrostatic (Figure 7c) and van der Waals (Figure 7d) intramolecular potentials for the whole protein did not differ significantly, except for during the 30–70 ns window, where the Ala-MnSOD displayed a lower electrostatic potential than the Val-MnSOD.

### 3.6. Secondary Structure

**Figure 8:**
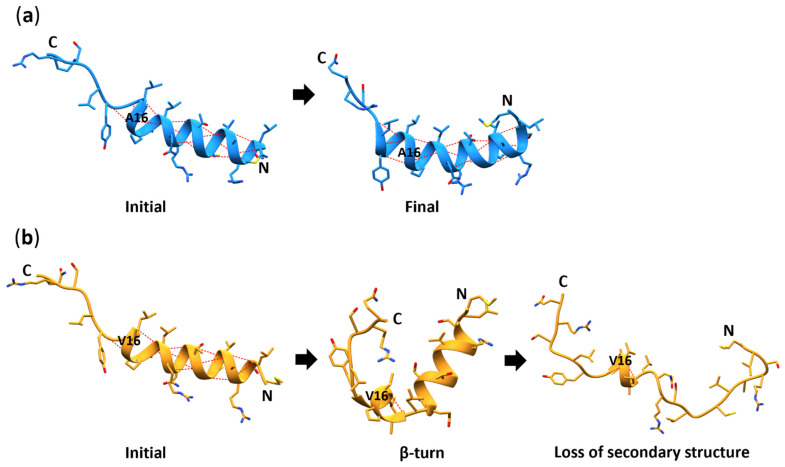
Cartoon and stick (residues 1–24) representations of the MTS during MD simulations. Initial and final structures are depicted for (**a**) the Ala-MTS, and with an additional transitional for (**b**) the Val-MTS. Blue and orange ribbons represent the secondary structure of the Ala- and Val-MTS, respectively. Side chains are depicted with sticks; blue/orange is for carbon, red for oxygen, dark blue for nitrogen, and yellow for sulfur. The red dashed lines denote intramolecular hydrogen bonds.

The secondary structure of the two variants was analyzed with VMD’s Timeline (Secondary Structure) plugin, which utilizes STRIDE. At the beginning of the simulations, both Ala- and Val-MTS formed a uniform α-helix extending between 2 and 16 for the Ala-MTS (Figure 8a) and between 3 and 16 for the Val-MTS (Figure 8b). However, at the end of 200 ns in MD simulations, the α-helical conformation extended between residues 2 and 20 for the Ala-MTS. In contrast, the α-helical conformation was preserved only between residues 13 and 17 for the Val-MTS. At ns 128, the Ala-MTS underwent an abrupt and temporary conversion to a 3_10_-helix between residues 10 and 14 for 2 ns. On the other hand, bending at residue 13 occurred at ns 40 for the Val-MTS, causing the collapse of the α-helical conformation, indicating a clear preference for the α-helical conformation in the case of the Ala-MTS.

## 4. Conclusions

This study aimed to decipher the unsolved structure of the MTS of the human MnSOD protein. A stable α-helix was observed for the Ala-MTS residues extending from residue 2 to 20. On the contrary, the α-helix of the Val-MTS collapsed shortly after the beginning of the simulation.

It is well established that an uninterrupted α-helix in the MTS of mitochondrial proteins facilitates their transport into mitochondria [29], implying that the Ala-MTS should be more effective for transporting the MnSOD precursor into mitochondria. This hypothesis is consistent with the study of Sutton, A. et al. [36], who showed that the Ala-MnSOD precursor produces up to 40% more of the active MnSOD homotetramer than the Val-MnSOD precursor. However, in that study, the functional activity was determined by examining intensity bands on activity gels, a method that has been criticized for possible accuracy limitations [75].

Another study by Sutton et al. showed that in human hepatoma cells HuH7 transfected with vectors encoding for the human Ala- or Val-MnSOD precursor, the Ala variant produced four-fold more mature exogenous protein and MnSOD activity compared to the Val variant [37]. Moreover, the results of our study are also partially consistent with the results of the study by Shimoda-Matsubayashi et al. [32], who proposed an α-helix between residues 10 and 17 of the Ala-MTS and a β-sheet for the Val-MTS. Another study, using I-Tasser [76] and ab initio modeling [77], suggested that valine disrupts the α-helix.

On the other hand, Bastakia, M. et al. [38] showed that the genotypes with the T allele (Val-MTS) confer higher enzymatic activity in human erythrocytes than the C allele (Ala-MTS). Two additional studies found increased levels of ROS-induced DNA damage biomarkers for the Ala/Val and Ala/Ala genotypes [47], and a greater oxidative damage from PAH exposure than Val/Val [48].

Our observations on the valine role in helical structures are supported by Mayer et al., Okamoto et al., and Chakrabartty et al. [78,79,80]. Moreover, Gregoret et al. and Tobias et al. demonstrated that alanine preserves the *α*-helix in contrast to valine, mainly due to a favorable helix-turn equilibrium observed in MD simulations of peptides [81,82]. A similar trend of alanine vs. valine in helix stability was also observed using homo-oligomeric decapeptidides subjected to a Monte Carlo simulated annealing [83]. As demonstrated by the experimental studies that employed circular dichroism, valine’s role in a helical structure is a result of the *β*-branching of the side chains [84,85,86,87], whereas hydrophobicity plays a negligible role [84,85]. These results are consistent with the observations from Lyu, P.C. and Jacchieri et al. [88,89]. They reported that branched side chains of isoleucine or valine are helix destabilizing, while linear side chain amino acids (e.g., alanine, 2-aminobutyric acid, norvaline, norleucine) are classified as helix stabilizing.

Another study that analyzed amino acid distributions in short, medium, and long helices with neighbor-dependent sequence analysis [90] found that Pro preceding Ala is 50% more common in short helices (four to seven residues) than in proteins. Notably, the MnSOD MTS contains a proline (Pro15) right before the variable residue 16, thus resulting in the Pro15-Ala16 sequence for the Ala-MTS and the Pro15-Val16 sequence for the Val-MTS. However, according to their classification, the MnSOD MTS falls under the long helices category (14 to 22 residues), for which no statistically significant trend between Pro and Ala or Val could be established. Nonetheless, the propensity values are still higher for Pro-Ala (0.52) than for Pro-Val (0.39), even in long helices [90]. Following Ala16 or Val16 in the MTS is leucine (Leu17). The study found a high propensity for the Ala-Leu sequence in helices (1.81) and a non-statistically significant propensity for the Val-Leu sequence (1.02) [90], which further supports our findings.

According to the presented computational data, the Ala-MTS prefers a uniform α-helix between residues 2 and 20, while the Val-MTS exhibits helical stability only between residues 13 and 17 for 200 ns. In conjunction with the established principles, the present results, therefore, suggest that the Ala-MTS variant is more easily transported into the mitochondria, resulting in a higher concentration of homotetrameric MnSOD than in the case of the Val-MTS.

The results show that the alanine variant retains the α-helix while the α-helix of the valine MTS variant collapses.

## Figures and Tables

**Table 1:** Comparison of the alanine to valine substitution resulting from SNP rs4880.

Rs4880	Alanine	Valine
Location	6q.25.3, SOD2 gene, position 47	
Nucleotide polymorphism	C (cytosine)	T (thymine)
Substitution location	Position 16 of MTS, position −9 of MnSOD (Figure 2a)	
Translates to	Alanine (Ala)	Valine (Val)
Protein secondary structure	(4–9) α-helix, (10–17) β-sheet [32]	(4–20) β-sheet [32]
Disease implications	Malignant pleural mesothelioma [42]	Heart disease in hereditary hemochromatosis [39]
	Acoustic neuroma [43]	An aggressive form of prostate cancer [40]
	Breast cancer [44]	Lung cancer [41]
	Intestinal metaplasia [45]	
	Gastric cancer [46]	
	Increased concentration of ROS-induced DNA damage biomarkers [47]	
	Greater oxidative injury caused by the PAH exposure [48]	

**Table 2:** The 10 most stable hydrogen bonds formed by the MTS with the rest of the protein or itself sorted based on their occupancy in %. Hydrogen bonds formed with the solvent or ions are not included. All hydrogen bonds formed by the MTS with the rest of the protein or itself are listed in the supplementary material Table S1.

Alanine			Valine		
Donor	Acceptor	Occupancy [%]	Donor	Acceptor	Occupancy [%]
Leu20 N	Ala16 O	43.5	Arg23 NH2	Glu66 OE2	36.3
Leu17 N	Leu13 O	39.6	Arg23 NH1	Glu66 OE1	35.9
Thr9 OG1	Ala5 O	31.3	Tyr69 OH	Gln12 O	35.1
Cys7 N	Ser3 O	29.7	Leu17 N	Leu13 O	25.4
Ser10 N	Val6 O	29.2	Arg23 NH2	Glu66 OE1	21.3
Thr9 N	Ala5 O	28.5	Arg23 NH1	Glu66 OE2	21.0
Leu13 N	Thr9 O	27.6	Asn63 ND2	Gln24 O	18.0
Arg23 NH1	Leu20 O	26.8	Cys7 N	Ser3 O	16.2
Gly8 N	Arg4 O	26.7	Thr9 OG1	Ala5 O	15.5
Arg23 NE	Asn63 OD1	25.8	Arg23 NH2	Asn63 OD1	13.5

## Data Availability

All data is contained within the article and supplementary material.

## Supplementary Materials

The following supporting information can be downloaded at: https://www.mdpi.com/article/10.3390/antiox11122348/s1, Table S1: The hydrogen bond analysis; Figure S1: The RMSD; Figure S2: The secondary structure comparison between different force fields along with the Psipred secondary structure prediction; Figure S3: A comparison of MTS sequences between various species; Movie S1: MD simulation video of the Ala-MnSOD variant around the time where the Val-MnSOD exhibits a helical breakdown; Movie S2: MD simulation video of the Val-MnSOD variant around the time where it exhibits a helical breakdown.

## Abbreviations

Ala	alanine
Ala-MTS	alanine variant of mitochondrial targeting sequence
Arg	arginine
Asn	asparagine
C_α_	alpha carbon atom in amino acids
C	cytosine allele
CC	cytosine-cytosine genotype for the RS4880 SNP
CT	cytosine-thymine genotype for the RS4880 SNP
ETC	electron transport chain
Gln	glutamine
Glu	glutamic acid
Gly	glycine
Leu	leucine
IMM	inner mitochondrial membrane
MD	molecular dynamics
Mn^3+^	manganese (III) ion
MnSOD	manganese superoxide dismutase
MPM	malignant pleural mesothelioma
MTS	mitochondrial targeting sequence
PAH	polycyclic aromatic hydrocarbon
Pro	proline
RGYR	radius of gyration
RMSD	root-mean-square deviation
RMSF	root-mean-square fluctuation
ROS	reactive oxygen species
SNP	single nucleotide polymorphism
SOD2	superoxide dismutase 2 gene
Val	valine
Val-MTS	valine variant of mitochondrial targeting sequence
T	thymine allele
TT	thymine-thymine genotype for the RS4880 SNP
Tyr	tyrosine
