# Supplementary material for: The Effect of the Ala16Val Mutation on the Secondary Structure of the Manganese Superoxide Dismutase Mitochondrial Targeting Sequence
Source: Antioxidants (Basel). 2022 Nov 27;11(12):2348. doi: 10.3390/antiox11122348 (PMC9774195; doi:10.3390/antiox11122348)
Supplement: Supplementary file 1 [file antioxidants-11-02348-s001.zip › antioxidants-1963731-supplementary.pdf]

antioxidants

MDPI

Supplementary File

Supplementary Information

for

The Effect of the Ala16Val Mutation on the Secondary Structure of the Manganese Superoxide Dismutase Mitochondrial Targeting Sequence

Matic Broz [1], Veronika Furlan [1], Samo Lešnik [1,2], Marko Jukič [1,3] and Urban Bren [1,2,3,*]

1 Faculty of Chemistry and Chemical Engineering, University of Maribor, Smetanova ulica 17, SI-2000 Maribor, Slovenia
2 Institute of Environmental Protection and Sensors, Beloruska ulica 7, SI-2000 Maribor, Slovenia
3 Faculty of Mathematics, Natural Sciences and Information Technologies, University of Primorska, Glagoljaška ulica 8, SI-6000 Koper, Slovenia
* Correspondence: urban.bren@um.si

Table S1 The most common hydrogen bonds formed by the MTS with the rest of the protein or itself sorted based on their occupancy. The hydrogen bonds formed with solvent or ions are not included.

	Alanine			Valine	
Donor	Acceptor	Occupancy [%]	Donor	Acceptor	Occupancy [%]
**Leu20 N**	**Ala16 O**	**43.50%**	Arg23 Nh2	Glu66 OE2	36.30%
Leu17 N	Leu13 O	39.60%	Arg23 Nh1	Glu66 OE	35.90%
Thr9 OG1	Ala5 O	31.30%	Tyr69 OH	Gln12 O	35.10%
Cys7 N	Ser3 O	29.70%	Leu17 N	Leu13 O	25.40%
Ser10 N	Val6 O	29.20%	Arg23 Nh2	Glu66 OE	21.30%
Thr9 N	Ala5 O	28.50%	Arg23 Nh1	Glu66 OE2	21.00%
Leu13 N	Thr9 O	27.60%	Asn63 Nd2	Gln24 O	18.00%
Arg23 NH1	Leu20 O	26.80%	Cys7 N	Ser3 O	16.20%
Gly8 N	Arg4 O	26.70%	Thr9 OG1	Ala5 O	15.50%
Arg23 Ne	Asn63 OD	25.80%	Arg23 NH2	Asn63 OD	13.50%
Arg23 NH2	Glu66 OE	25.40%	Val6 N	Leu2 O	10.60%
Arg23 NH1	Glu66 OE	23.90%	Gly8 N	Arg4 O	10.60%
Arg23 NH1	Glu66 OE2	23.80%	Thr9 N	Ala5 O	9.80%
Arg23 NH2	Glu66 OE2	22.80%	Leu13 N	Thr9 O	8.40%
Arg11 N	Cys7 O	17.20%	Ser10 OG	Val6 O	7.90%
Ser10 OG	Val6 O	16.80%	Gln12 NE2	Gln81 OE	7.90%
Val6 N	Leu2 O	16.40%	Ser10 N	Val6 O	6.50%
Tyr19 N	Pro15 O	16.10%	Leu2 N	Gln70 OE	4.50%
Gly21 N	Leu17 O	14.20%	Ser10 OG	Cys7 O	4.00%
Gln81 NE2	Thr9 OG1	12.20%	Gly18 N	Ala14 O	3.80%
Gln12 N	Gly8 O	11.90%	Arg11 N	Cys7 O	3.60%
Arg23 NH2	Asn63 OD	9.70%	Gln24 NE2	Hsd26 O	3.50%

Arg23 NH1	Gly21 O	6.10%	Ser22 OG	Gln24 OE	3.30%
Gln12 NE2	Gly8 O	4.60%	Gln12 N	Thr9 O	3.00%
Ala14 N	Ser10 O	4.40%	Hsd95 Nd	Leu20 O	2.70%
Lys25 Nz	Arg23 O	4.30%	Gln12 NE2	Gln85 OE	2.30%
Thr9 OG1	Gln81 OE	4.00%	Gln12 N	Gly8 O	2.20%
Ser27 OG	Gln24 O	3.90%	Gln12 NE2	Gly8 O	1.90%
Gln85 NE2	Gln12 OE	3.60%	Ala14 N	Ser10 O	1.90%
**Ala16 N**	**Gln12 O**	**2.60%**	Gln85 NE2	Gln12 OE	1.80%
Hsd26 N	Gln24 OE	2.20%	Arg11 NH1	Arg11 O	1.70%
Gln24 N	Gln24 OE	1.70%	Ser27 OG	Gln24 O	1.40%
Leu28 N	Gln24 OE	1.30%	Ser22 N	Hsd95 NE2	1.20%
Arg11 NH1	Arg11 O	1.30%	Gly8 N	Ala5 O	1.20%
Gln24 NE2	Hsd26 O	1.20%	**Val16 N**	**Leu13 O**	**1.00%**
Ala5 N	Leu2 O	0.80%	Gln24 NE2	Ser27 O	1.00%
Gln24 NE2	Ser27 O	0.80%	Gln24 NE2	Arg23 O	0.90%
Arg23 NH2	Glu66 CD	0.70%	Gln24 N	Ser22 OG	0.80%
Ser10 OG	Cys7 O	0.70%	Hsd26 N	Gln24 OE	0.80%
Ser27 N	Gln24 O	0.60%	Lys25 N	Gln24 OE	0.80%
Gly18 N	Ala14 O	0.60%	Gln24 NE2	Ser22 OG	0.70%
Gln85 NE2	Gln12 O	0.60%	Ala5 N	Leu2 O	0.70%
Tyr69 OH	Gln12 O	0.60%	Met1 N	Gln70 OE	0.70%
Gln81 NE2	Thr9 O	0.50%	Gln24 NE2	Hsd55 NE2	0.70%
Ser22 OG	Ser27 OG	0.40%	Tyr19 N	Pro15 O	0.60%
Lys25 N	Gln24 OE	0.40%	Ser3 OG	Gln70 O	0.60%
Ala5 N	Met1 O	0.40%	Arg11 N	Gln81 OE	0.60%
Gln24 NE2	Leu28 O	0.40%	Arg11 Ne	Gln12 OE	0.50%
Ser27 N	Gln24 OE	0.40%	Met1 N	Ala74 O	0.50%
Gln12 N	Thr9 O	0.40%	Leu13 N	Ser10 O	0.50%
Ser27 OG	Ser22 OG	0.30%	Cys7 N	Arg4 O	0.50%
**Tyr19 N**	**Ala16 O**	**0.30%**	Gln12 N	Gln81 OE	0.50%
Arg4 NH1	Arg4 O	0.30%	Ser22 OG	Gln24 O	0.40%
Arg4 Ne	Arg4 O	0.30%	Gln81 NE2	Thr9 OG1	0.40%
Gln12 NE2	Gln12 O	0.30%	Ser10 OG	Gln70 OE	0.40%
Gln12 NE2	Gln81 OE	0.30%	Gln12 NE2	Arg11 O	0.40%
Gln24 NE2	Ser22 O	0.20%	Leu17 N	Ala14 O	0.40%
Ser22 OG	Gln24 OE	0.20%	Arg4 NH1	Ser3 O	0.40%
Gln171 NE2	Gly18 O	0.20%	Gln81 NE2	Thr9 O	0.40%
Gly18 N	Pro15 O	0.20%	Gly8 N	Ala74 O	0.40%
Leu20 N	Leu17 O	0.20%	Tyr69 OH	Ser10 OG	0.40%
Leu28 N	Gln24 O	0.20%	Arg23 NH1	Gly21 O	0.30%
Asn63 Nd2	Gln24 O	0.20%	Arg23 NH1	Leu20 O	0.30%
Gln24 NE2	Asp30 OD	0.20%	Lys25 Nz	Ser22 O	0.30%
Tyr19 OH	Gln85 OE	0.10%	**Tyr19 N**	**Val16 O**	**0.30%**
Gln171 NE2	Tyr19 O	0.10%	Val6 N	Ser3 O	0.30%
Gln24 N	Ser22 OG	0.10%	Gln24 NE2	Ser22 O	0.30%
Gln24 NE2	Leu20 O	0.10%	Arg11 NH1	Gln12 OE	0.30%
Gln24 NE2	Ser22 OG	0.10%	Thr9 OG1	Gln12 OE	0.30%
Met1 N	Lys75 O	0.10%	Ser3 N	Gln70 OE	0.30%
Ser27 OG	Ser22 O	0.10%	Arg11 NH1	Thr9 O	0.30%
Met1 N	Ala74 O	0.10%	Ala5 N	Met1 O	0.20%
Ser3 OG	Leu2 O	0.10%	**Arg23 NH2**	**Val16 O**	**0.20%**

Ser3 OG	Gly76 O	0.10%	Arg11 NH1	Ser10 OG	0.20%
Arg4 NH1	Ser3 OG	0.10%	Gln70 NE2	Val6 O	0.20%
Lys89 Nz	Tyr19 OH	0.10%	Arg23 N	Hsd95 NE2	0.20%
Gln24 NE2	Hsd26 NE2	0.10%	Arg4 NH1	Ser3 OG	0.20%
Gln12 NE2	Gln85 OE	0.10%	Gln12 N	Thr9 OG1	0.20%
Cys7 Sg	Ser3 O	0.10%	Gln12 NE2	Thr9 OG1	0.20%
Gln24 NE2	Asp30 OD2	0.10%	Met1 N	Glu71 OE	0.20%
Asp30 N	Gln24 OE	0.10%	Gln81 NE2	Gln12 OE	0.20%
Ser27 OG	Arg23 O	0.10%	Arg11 N	Thr9 OG1	0.20%
Ala14 N	Arg11 O	0.10%	Tyr19 OH	Ser22 OG	0.20%
Hsd26 Nd	Gln24 OE	0.10%	Arg4 NH1	Ala74 O	0.20%
Gln24 NE2	Arg23 O	0.10%	Met1 N	Glu67 OE	0.20%
			ARG4 NE	LEU73 O	0.20%
			ARG4 NE	VAL6 O	0.20%
			SER3 N	LYS75 O	0.20%
			THR9 OG1	GLY8 O	0.20%
			SER10 OG	GLN12 O	0.20%
			ARG23 NH2	ASP30 OD	0.10%
			GLN171 NE2	LEU20 O	0.10%
			TYR19 OH	GLN85 OE	0.10%
			GLN24 NE2	SER27 OG	0.10%
			SER22 OG	HSD95 NE2	0.10%
			ARG4 NH2	SER3 OG	0.10%
			LYS25 N	SER22 OG	0.10%
			LYS25 NZ	ARG23 O	0.10%
			GLN85 NE2	GLN12 O	0.10%
			TYR69 OH	GLN12 OE	0.10%
			LYS89 NZ	GLY18 O	0.10%
			SER27 N	GLN24 OE	0.10%
			GLN24 NE2	HSD26 NE2	0.10%
			SER22 OG	HSD26 NE2	0.10%
			GLN70 NE2	SER10 OG	0.10%
			ARG23 NH1	GLU66 CD	0.10%
			MET1 N	GLU71 OE2	0.10%
			GLN12 NE2	CYS7 O	0.10%
			HSD95 CE	SER22 O	0.10%
			CYS7 SG	SER3 O	0.10%
			SER22 OG	HSD55 NE2	0.10%
			GLN24 NE2	GLN24 O	0.10%
			ARG11 NH2	SER10 OG	0.10%
			LEU28 N	GLN24 OE	0.10%
			ARG4 NH2	ALA74 O	0.10%
			MET1 N	GLU67 OE2	0.10%
			GLN12 NE2	TYR69 OH	0.10%
			ARG4 NH1	GLN70 OE	0.10%
			ALA14 N	ARG11 O	0.10%
			CYS7 SG	ARG4 O	0.10%
			ARG4 N	GLY76 O	0.10%
			HSD95 ND	SER22 O	0.10%
			ARG4 NH1	VAL6 O	0.10%
			HSD26 ND	GLN24 OE	0.10%

THR9 OG1	CYS7 O	0.10%
CYS7 N	GLY76 O	0.10%
ARG11 NH2	GLN12 OE	0.10%
ARG4 NE	MET1 O	0.10%
ARG11 NE	THR9 OG1	0.10%
GLY18 N	PRO15 O	0.10%
GLY8 N	LEU73 O	0.10%
SER10 OG	GLN81 OE	0.10%

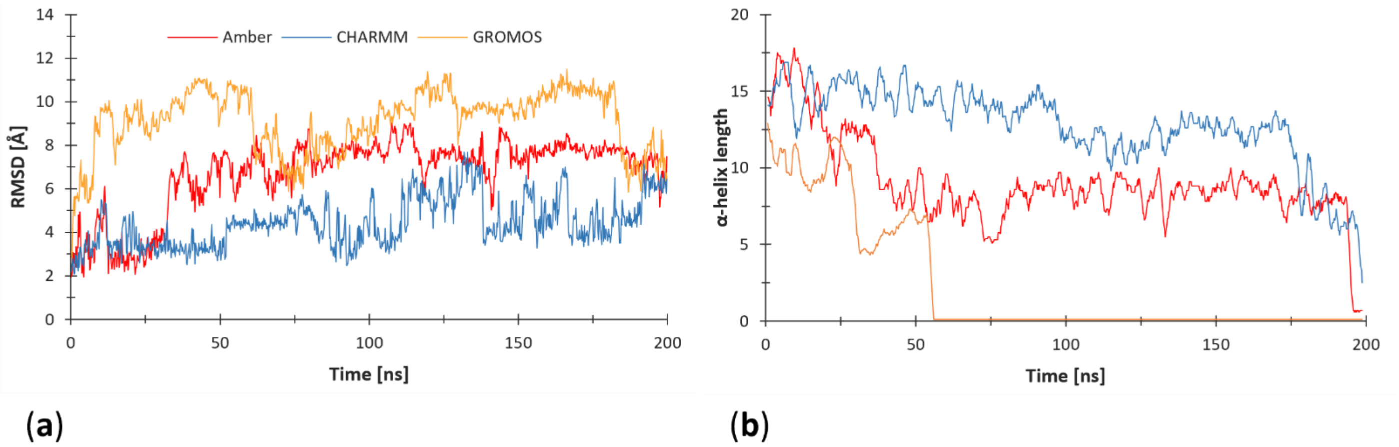

Figure S1 A graphical comparison of the PEP-FOLD3 Val-MTS model in three different force fields: Amber, CHARMM, and GROMOS. The graphs depict (**a**) RMSD values and (**b**) the lengths of the α-helix.

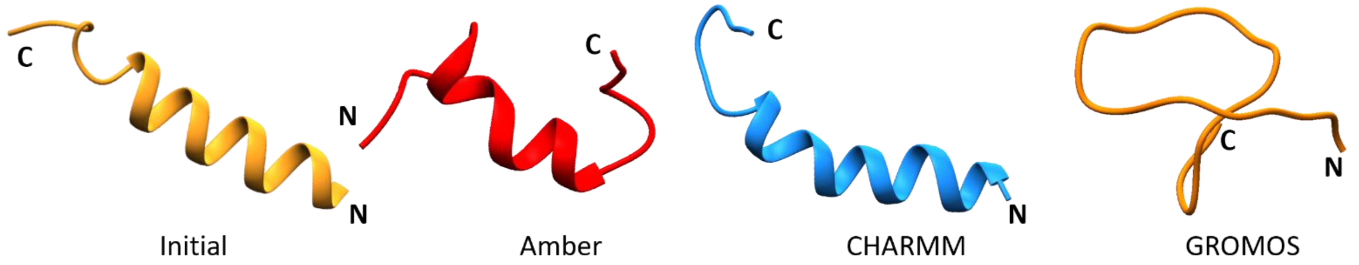

Figure S2 A cartoon representations of the initial PEP-FOLD3 Val-MTS structure with the most common structural clusters in MD simulations using Amber, CHARMM, and GROMOS force fields.

```
1   5    10       16   20  24
MLSRAVCGTSRQLAP | V | LGYLGSRQ – Homo sapiens (P04179)
MLSRAVCGTSRQLAP | A | LGYLGSRQ – Pan troglodytes (A0A2I3T2J1)
MLSRAVCGTSRQLAP | A | LGYLGSRQ – Pan paniscus A0A2R9BMB0
MLSRAVCGTGRQLAP | A | LGYLGSRQ – Macaca nemestrina (Q5FB30)
MLSRAVCGTGRQLAP | A | LGYLGSRQ – Rhinopithecus roxellana (A0A2K6NUA4)
MLSRAVCGTGRQLAP | A | LGYLGSRQ – Macaca fascicularis (Q8HXP3)
MLSRAVCGTGRQLAP | A | LGYLGSRQ – Cebus imitator (A0A2K5QGB9)
MLSRAVCGTRRQLAP | A | LGYLGSRQ – Macaca nemestrina (A0A2K6BV32)
MLSRAVCGTGRQLAL | A | LGYLGSRQ – Aotus nancymaae (A0A2K5CX40)
MLSRAACSTSRKLVP | A | LGVLGSRQ – Ovis aries (C8BKD6)
MTARAY   TSRQLAP | A | LGYLGSRQ – Mandrillus leucophaeus (A0A2K5YFD5)
MLSRAACSAGARLAP | A | LGALGCRQ – Spermophilus tridecemlineatus (I3MC61)
ML   AS   TSRQLAP | A | LGYLGSRQ – Western lowland gorilla (G3QYT5)
MLSRAACSTSRRLVP | A | LSVLGSRQ – Bos taurus (P41976)
MLSRAALSSSRTLVP | A | LGALGSRQ – Panthera leo (A0A8C8WJT4)
MLCRVMSRRTSKLVP | A | LGCLGSRQ – Monodelphis domestica (F7GJS0)
MLCRAVCSASRRLAP | A | LGILGVRQ – Cavia porcellus (P49114)
MLCRAACSTSRKLVP | A | LGSLGSRQ – Equus caballus (Q9XS41)
MLCRAACSAGRRLGP | A | ASTAGSRH – Rattus norvegicus (P07895)
MLCRAACSTGRRLGP | V | AGAAGSRH – Mus musculus (P09671)
MLPYAACSASRRLAP | A | LGILGVRQ – Cavia porcellus (P49114)
MLCRVASSAGRSLSP | A | LGALGSRQ – Castor canadensis (A0A250YCN7)
MLCRAACSAGGRLAP | A | ATAAGCRQ – Mesocricetus auratus (A0A1U7QH60)
MLCRLSSAGRSSVKV | V | APLGCLAS – Melopsittacus undulatus (Q802D9)
```

Figure S3 A comparison of MTS sequences between various species. Red letters represent amino acids different from the *Homo sapiens* MTS sequence. Bolded letters represent the Ala16Val substitution in the *Homo sapiens* genome, which is scarcely present in other species' genomes.

Psipred secondary structure prediction

ALA variant

job,step,type,name,version,parameters

psipred,0,Dataset,uniref90,latest,"null"

psipred,0,Dataset,hhdb uniclust30,2018_08,"null"

psipred,0,Software,psiblast,2.2.26,"-a 2 -b 0 -j 20 -h 0.01 -I F"

psipred,0,Software,hhblits,2.0.16,"-iterations 3 -diff inf -cov 10 -Z 10000 -B 10000 -maxfilt 10000 -maxmem 5 -norealign"

psipred,1,Software,psipred,4.1,"null"

psipred,2,Software,psipass2,4.1,"null"

PSIPRED VFORMAT (PSIPRED V4.0)

1 M C	0.999	0.001	0.000
2 L C	0.633	0.371	0.018
3 S H	0.249	0.713	0.013
4 R H	0.141	0.838	0.016
5 A H	0.148	0.837	0.009
6 V H	0.270	0.731	0.005
7 C H	0.272	0.720	0.006
8 G H	0.200	0.793	0.004
9 T H	0.160	0.828	0.003
10 S H	0.101	0.891	0.005
11 R H	0.333	0.658	0.003
12 Q H	0.223	0.762	0.004
13 L H	0.193	0.798	0.004
14 A H	0.124	0.863	0.005
15 P H	0.068	0.927	0.005
**16 A H**	**0.155**	**0.839**	**0.007**
17 L H	0.394	0.609	0.008
18 G H	0.361	0.634	0.013
19 Y H	0.149	0.840	0.027
20 L H	0.223	0.769	0.021
21 G H	0.462	0.545	0.012
22 S C	0.668	0.345	0.017
23 R C	0.767	0.212	0.026
24 Q C	0.895	0.089	0.027
25 K C	0.859	0.055	0.067
26 H C	0.544	0.065	0.387
27 S C	0.606	0.022	0.373
28 L C	0.948	0.008	0.039
29 P C	0.986	0.007	0.011
30 D C	0.974	0.015	0.007
31 L C	0.982	0.010	0.005
32 P C	0.988	0.005	0.004
33 Y C	0.979	0.010	0.005
34 D C	0.977	0.012	0.004
35 Y C	0.746	0.277	0.004
36 G C	0.833	0.191	0.007
37 A C	0.827	0.190	0.015
38 L C	0.820	0.201	0.018
39 E C	0.590	0.376	0.016
40 P C	0.537	0.379	0.032
41 H C	0.663	0.269	0.086

42 I C	0.424	0.393	0.224
43 N C	0.903	0.070	0.020
44 A H	0.043	0.950	0.001
45 Q H	0.022	0.978	0.001
46 I H	0.028	0.973	0.001
47 M H	0.030	0.971	0.002
48 Q H	0.073	0.927	0.004
49 L H	0.061	0.939	0.007
50 H H	0.170	0.828	0.005
51 H H	0.387	0.600	0.003
52 S H	0.079	0.914	0.003
53 K H	0.051	0.948	0.003
54 H H	0.094	0.898	0.003
55 H H	0.034	0.965	0.002
56 A H	0.011	0.990	0.001
57 A H	0.010	0.991	0.001
58 Y H	0.022	0.978	0.001
59 V H	0.033	0.968	0.001
60 N H	0.045	0.954	0.001
61 N H	0.046	0.952	0.001
62 L H	0.015	0.985	0.001
63 N H	0.009	0.991	0.000
64 V H	0.007	0.993	0.000
65 T H	0.005	0.995	0.000
66 E H	0.010	0.990	0.000
67 E H	0.010	0.990	0.000
68 K H	0.009	0.992	0.000
69 Y H	0.008	0.993	0.000
70 Q H	0.005	0.996	0.000
71 E H	0.004	0.996	0.000
72 A H	0.006	0.995	0.000
73 L H	0.017	0.984	0.001
74 A H	0.084	0.915	0.002
75 K C	0.799	0.231	0.000
76 G C	0.989	0.011	0.000
77 D C	0.978	0.019	0.001
78 V H	0.216	0.783	0.001
79 T H	0.095	0.895	0.003
80 A H	0.024	0.976	0.001
81 Q H	0.025	0.974	0.002
82 I H	0.025	0.975	0.002
83 A H	0.044	0.957	0.002

84 L H	0.077	0.924	0.001
85 Q H	0.075	0.925	0.001
86 P H	0.029	0.970	0.002
87 A H	0.050	0.951	0.002
88 L H	0.034	0.965	0.003
89 K H	0.149	0.845	0.005
90 F H	0.438	0.562	0.010
91 N C	0.747	0.225	0.017
92 G C	0.812	0.171	0.032
93 G C	0.779	0.223	0.034
94 G C	0.569	0.338	0.041
95 H C	0.602	0.337	0.047
96 I H	0.363	0.561	0.038
97 N H	0.235	0.713	0.032
98 H C	0.639	0.269	0.029
99 S C	0.472	0.446	0.048
100 I H	0.201	0.723	0.046
101 F H	0.229	0.691	0.057
102 W C	0.753	0.224	0.013
103 T C	0.539	0.410	0.021
104 N C	0.731	0.238	0.014
105 L C	0.919	0.086	0.015
106 S C	0.983	0.008	0.008
107 P C	0.981	0.010	0.007
108 N C	0.959	0.027	0.006
109 G C	0.964	0.022	0.005
110 G C	0.968	0.022	0.006
111 G C	0.972	0.014	0.009
112 E C	0.976	0.008	0.012
113 P C	0.982	0.006	0.008
114 K C	0.978	0.013	0.003
115 G H	0.300	0.703	0.001
116 E H	0.063	0.932	0.003
117 L H	0.010	0.990	0.000
118 L H	0.006	0.994	0.000
119 E H	0.003	0.997	0.000
120 A H	0.004	0.997	0.000
121 I H	0.005	0.995	0.000
122 K H	0.006	0.995	0.000
123 R H	0.018	0.983	0.001
124 D H	0.064	0.936	0.002
125 F H	0.432	0.581	0.001

126 G C	0.940	0.081	0.001
127 S C	0.974	0.025	0.002
128 F H	0.020	0.980	0.000
129 D H	0.015	0.985	0.001
130 K H	0.007	0.994	0.000
131 F H	0.004	0.996	0.000
132 K H	0.003	0.997	0.000
133 E H	0.003	0.997	0.000
134 K H	0.003	0.997	0.000
135 L H	0.006	0.994	0.000
136 T H	0.010	0.991	0.000
137 A H	0.010	0.991	0.000
138 A H	0.024	0.979	0.001
139 S H	0.035	0.966	0.002
140 V H	0.109	0.896	0.003
141 G H	0.229	0.775	0.014
142 V C	0.806	0.142	0.011
143 Q C	0.879	0.061	0.043
144 G C	0.886	0.028	0.065
145 S C	0.941	0.014	0.044
146 G C	0.697	0.026	0.274
147 W E	0.089	0.005	0.860
148 G E	0.023	0.002	0.949
149 W E	0.021	0.002	0.934
150 L E	0.017	0.001	0.931
151 G E	0.014	0.001	0.964
152 F E	0.014	0.003	0.944
153 N E	0.091	0.006	0.893
154 K C	0.966	0.010	0.020
155 E C	0.983	0.011	0.006
156 R C	0.981	0.013	0.004
157 G C	0.984	0.004	0.021
158 H E	0.392	0.006	0.591
159 L E	0.041	0.003	0.955
160 Q E	0.019	0.002	0.977
161 I E	0.013	0.001	0.947
162 A E	0.013	0.002	0.942
163 A E	0.084	0.008	0.872
164 C C	0.843	0.010	0.123
165 P C	0.951	0.027	0.023
166 N C	0.977	0.013	0.007
167 Q C	0.949	0.033	0.013

168	D C	0.963	0.019	0.015
169	P C	0.944	0.032	0.018
170	L C	0.904	0.071	0.024
171	Q C	0.875	0.080	0.028
172	G C	0.863	0.139	0.020
173	T C	0.878	0.137	0.018
174	T C	0.927	0.061	0.018
175	G C	0.964	0.015	0.023
176	L C	0.888	0.014	0.117
177	I C	0.588	0.008	0.413
178	P E	0.219	0.009	0.754
179	L E	0.147	0.012	0.822
180	L E	0.051	0.005	0.891
181	G E	0.035	0.003	0.914
182	I E	0.064	0.010	0.870
183	D E	0.194	0.020	0.725
184	V H	0.446	0.534	0.049
185	W H	0.270	0.805	0.036
186	E H	0.291	0.697	0.009
187	H C	0.829	0.171	0.012
188	A H	0.391	0.603	0.006
189	Y H	0.412	0.574	0.011
190	Y H	0.161	0.826	0.008
191	L H	0.170	0.819	0.009
192	Q H	0.425	0.561	0.014
193	Y C	0.719	0.274	0.014
194	K C	0.849	0.145	0.015
195	N C	0.872	0.137	0.009
196	V C	0.922	0.102	0.003
197	R C	0.974	0.026	0.002
198	P H	0.065	0.933	0.000
199	D H	0.064	0.933	0.003
200	Y H	0.014	0.986	0.001
201	L H	0.007	0.993	0.001
202	K H	0.014	0.987	0.001
203	A H	0.022	0.979	0.002
204	I H	0.047	0.955	0.003
205	W H	0.028	0.974	0.013
206	N H	0.124	0.853	0.048
207	V C	0.472	0.414	0.050
208	I C	0.531	0.392	0.071
209	N C	0.955	0.036	0.014

210	W H	0.034	0.963	0.001
211	E H	0.022	0.978	0.001
212	N H	0.011	0.989	0.000
213	V H	0.004	0.996	0.000
214	T H	0.003	0.997	0.000
215	E H	0.003	0.997	0.000
216	R H	0.005	0.996	0.000
217	Y H	0.010	0.992	0.000
218	M H	0.011	0.990	0.000
219	A H	0.043	0.960	0.001
220	C H	0.145	0.859	0.004
221	K H	0.457	0.550	0.004
222	K C	0.999	0.000	0.000

```
# PSIPRED HFORMAT (PSIPRED V4.0)

Conf: 924664456735678622650358712999999946662130899998862888999999
Pred: CCHHHHHHHHHHHHHHHHHHHCCCCCCCCCCCCCCCCCCCCCCHHHHHHHHHHHHHHHHH
  AA: MLSRAVCGTSRQLAPALGYLGSRQKHSLPDLPYDYGALEPHINAQIMQLHHSKHHAAYVN
              10        20        30        40        50        60

Conf: 999999999999985995899998899961565221430545148999999999489999
Pred: HHHHHHHHHHHHHHCCCHHHHHHHHHHHHHCCCCHHCCHHCCCCCCCCCCCCCCHHHHHH
  AA: NLNVTEEKYQEALAKGDVTAQIALQPALKFNGGGHINHSIFWTNLSPNGGGEPKGELLEA
              70        80        90       100       110       120

Conf: 999818999999999999975688847999998999919999779999987778971568
Pred: HHHHHCCHHHHHHHHHHHHHHCCCCCEEEEEEECCCCEEEEEECCCCCCCCCCCCCCEEE
  AA: IKRDFGSFDKFKEKLTAASVGVQGSGWGWLGFNKERGHLQIAACPNQDPLQGTTGLIPLL
             130       140       150       160       170       180

Conf: 885054621661477898899999970199999999999709
Pred: EEEHHHCHHHHHCCCCCHHHHHHHHHCCCHHHHHHHHHHHHC
  AA: GIDVWEHAYYLQYKNVRPDYLKAIWNVINWENVTERYMACKK
             190       200       210       220
```

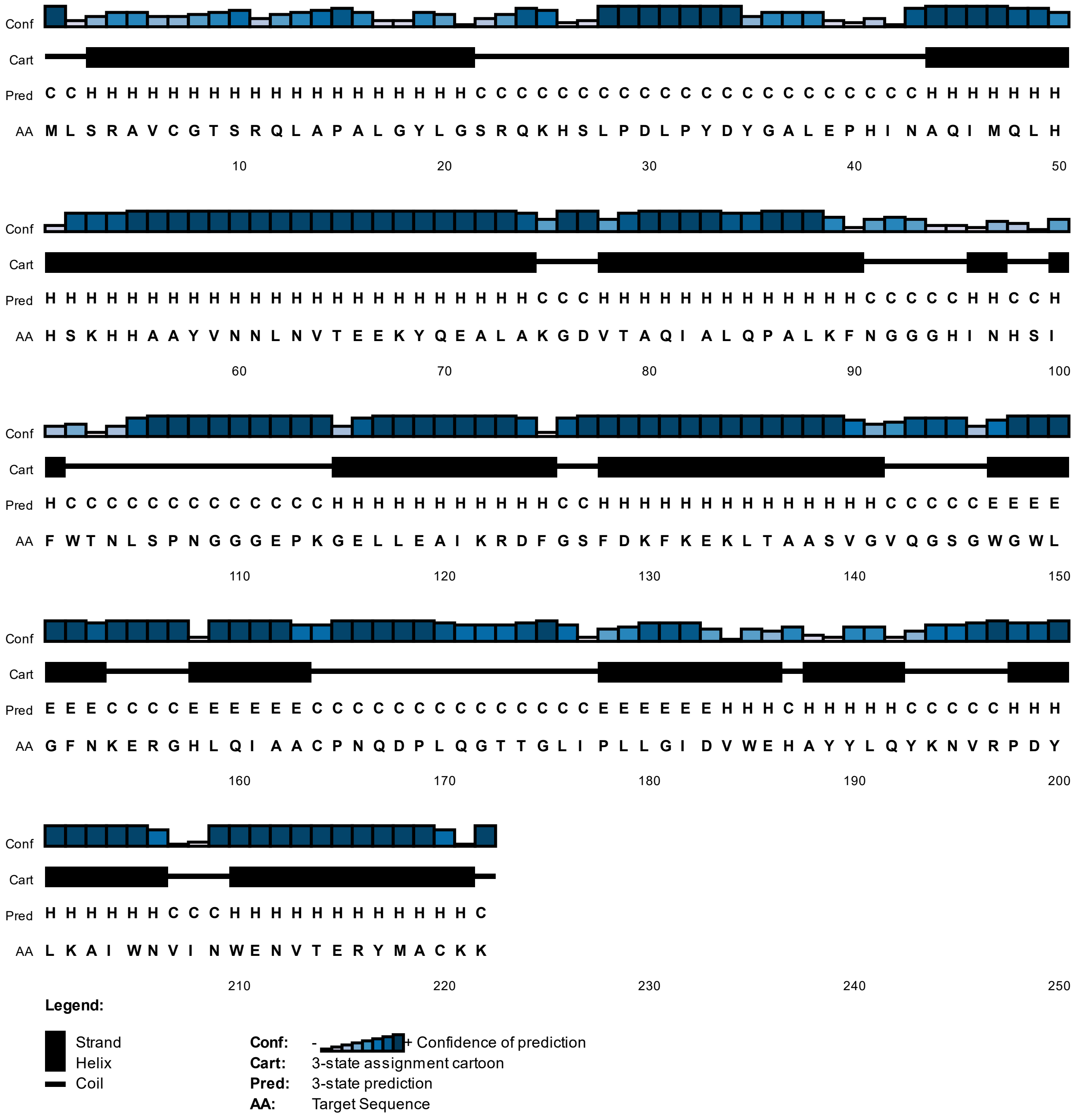

VAL variant

job,step,type,name,version,parameters

psipred,0,Dataset,uniref90,latest,"null"

psipred,0,Dataset,hhdb uniclust30,2018_08,"null"

psipred,0,Software,psiblast,2.2.26,"-a 2 -b 0 -j 20 -h 0.01 -I F"

psipred,0,Software,hhblits,2.0.16,"-iterations 3 -diff inf -cov 10 -Z 10000 -B 10000 -maxfilt 10000 -maxmem 5 -norealign"

psipred,1,Software,psipred,4.1,"null"

psipred,2,Software,psipass2,4.1,"null"

PSIPRED VFORMAT (PSIPRED V4.0)

```
 1 M C   0.999  0.001  0.000
 2 L C   0.660  0.349  0.014
 3 S H   0.229  0.744  0.009
 4 R H   0.125  0.858  0.012
 5 A H   0.131  0.854  0.006
 6 V H   0.146  0.854  0.004
 7 C H   0.117  0.878  0.004
 8 G H   0.121  0.873  0.003
 9 T H   0.089  0.904  0.003
10 S H   0.098  0.896  0.005
11 R H   0.348  0.642  0.003
12 Q H   0.167  0.821  0.004
13 L H   0.163  0.827  0.004
14 A H   0.129  0.856  0.005
15 P H   0.064  0.932  0.006
16 V H   0.093  0.902  0.010
17 L H   0.196  0.798  0.018
18 G H   0.253  0.731  0.017
19 Y H   0.147  0.838  0.027
20 L H   0.217  0.777  0.023
21 G H   0.457  0.549  0.011
22 S C   0.664  0.348  0.016
23 R C   0.758  0.211  0.028
24 Q C   0.874  0.097  0.035
25 K C   0.846  0.057  0.073
26 H C   0.512  0.059  0.418
27 S C   0.585  0.021  0.395
28 L C   0.947  0.008  0.040
29 P C   0.985  0.007  0.011
30 D C   0.974  0.015  0.007
31 L C   0.982  0.010  0.005
32 P C   0.988  0.005  0.004
33 Y C   0.979  0.010  0.005
34 D C   0.977  0.012  0.004
```

35 Y C	0.750	0.274	0.004
36 G C	0.842	0.184	0.007
37 A C	0.834	0.184	0.014
38 L C	0.818	0.204	0.018
39 E C	0.563	0.402	0.015
40 P C	0.506	0.415	0.030
41 H C	0.648	0.282	0.086
42 I C	0.431	0.383	0.226
43 N C	0.901	0.070	0.021
44 A H	0.043	0.950	0.001
45 Q H	0.023	0.976	0.002
46 I H	0.029	0.972	0.001
47 M H	0.032	0.970	0.002
48 Q H	0.076	0.924	0.004
49 L H	0.062	0.937	0.008
50 H H	0.167	0.830	0.005
51 H H	0.381	0.605	0.003
52 S H	0.079	0.914	0.003
53 K H	0.050	0.949	0.003
54 H H	0.094	0.898	0.003
55 H H	0.035	0.963	0.002
56 A H	0.011	0.989	0.001
57 A H	0.010	0.991	0.001
58 Y H	0.021	0.980	0.001
59 V H	0.030	0.971	0.001
60 N H	0.039	0.961	0.001
61 N H	0.045	0.954	0.001
62 L H	0.014	0.986	0.001
63 N H	0.009	0.992	0.000
64 V H	0.007	0.993	0.000
65 T H	0.005	0.995	0.000
66 E H	0.011	0.990	0.000
67 E H	0.010	0.990	0.000
68 K H	0.009	0.992	0.000
69 Y H	0.008	0.993	0.000
70 Q H	0.005	0.996	0.000
71 E H	0.004	0.996	0.000
72 A H	0.006	0.995	0.000
73 L H	0.017	0.984	0.001
74 A H	0.085	0.914	0.002
75 K C	0.801	0.227	0.000
76 G C	0.989	0.011	0.000

77 D C	0.978	0.018	0.001
78 V H	0.226	0.774	0.001
79 T H	0.100	0.888	0.003
80 A H	0.025	0.975	0.001
81 Q H	0.025	0.974	0.002
82 I H	0.024	0.976	0.002
83 A H	0.043	0.958	0.002
84 L H	0.077	0.923	0.001
85 Q H	0.092	0.907	0.001
86 P H	0.031	0.969	0.002
87 A H	0.052	0.950	0.002
88 L H	0.036	0.963	0.003
89 K H	0.145	0.849	0.005
90 F H	0.439	0.561	0.011
91 N C	0.745	0.225	0.017
92 G C	0.813	0.170	0.032
93 G C	0.787	0.215	0.034
94 G C	0.586	0.325	0.040
95 H C	0.622	0.320	0.046
96 I H	0.373	0.554	0.037
97 N H	0.228	0.724	0.030
98 H C	0.634	0.269	0.029
99 S C	0.505	0.427	0.045
100 I H	0.189	0.738	0.042
101 F H	0.235	0.687	0.053
102 W C	0.770	0.212	0.012
103 T C	0.550	0.405	0.020
104 N C	0.727	0.245	0.013
105 L C	0.916	0.091	0.015
106 S C	0.984	0.008	0.008
107 P C	0.982	0.009	0.007
108 N C	0.959	0.028	0.006
109 G C	0.964	0.023	0.005
110 G C	0.969	0.021	0.006
111 G C	0.973	0.014	0.008
112 E C	0.976	0.007	0.011
113 P C	0.982	0.006	0.008
114 K C	0.978	0.013	0.003
115 G H	0.312	0.691	0.001
116 E H	0.066	0.928	0.003
117 L H	0.011	0.990	0.000
118 L H	0.006	0.994	0.000

119 E H	0.003	0.997	0.000
120 A H	0.004	0.997	0.000
121 I H	0.005	0.996	0.000
122 K H	0.006	0.995	0.000
123 R H	0.018	0.984	0.001
124 D H	0.060	0.940	0.002
125 F H	0.420	0.592	0.001
126 G C	0.948	0.070	0.001
127 S C	0.975	0.023	0.002
128 F H	0.020	0.981	0.000
129 D H	0.015	0.985	0.001
130 K H	0.007	0.994	0.000
131 F H	0.004	0.996	0.000
132 K H	0.003	0.997	0.000
133 E H	0.003	0.997	0.000
134 K H	0.003	0.997	0.000
135 L H	0.006	0.994	0.000
136 T H	0.010	0.991	0.000
137 A H	0.010	0.991	0.000
138 A H	0.023	0.980	0.001
139 S H	0.034	0.967	0.002
140 V H	0.113	0.891	0.003
141 G H	0.223	0.782	0.013
142 V C	0.808	0.143	0.010
143 Q C	0.882	0.061	0.040
144 G C	0.888	0.027	0.064
145 S C	0.939	0.015	0.045
146 G C	0.686	0.029	0.280
147 W E	0.090	0.005	0.857
148 G E	0.023	0.002	0.948
149 W E	0.021	0.002	0.933
150 L E	0.017	0.001	0.930
151 G E	0.014	0.001	0.964
152 F E	0.014	0.003	0.943
153 N E	0.092	0.006	0.892
154 K C	0.966	0.010	0.021
155 E C	0.983	0.011	0.007
156 R C	0.981	0.013	0.004
157 G C	0.984	0.004	0.021
158 H E	0.370	0.006	0.615
159 L E	0.039	0.003	0.957
160 Q E	0.019	0.001	0.977

161	I E	0.013	0.001	0.947
162	A E	0.013	0.002	0.942
163	A E	0.084	0.008	0.869
164	C C	0.824	0.010	0.137
165	P C	0.945	0.029	0.025
166	N C	0.976	0.014	0.008
167	Q C	0.948	0.034	0.014
168	D C	0.962	0.019	0.015
169	P C	0.944	0.033	0.018
170	L C	0.905	0.070	0.024
171	Q C	0.878	0.079	0.027
172	G C	0.866	0.138	0.020
173	T C	0.874	0.140	0.019
174	T C	0.926	0.061	0.018
175	G C	0.963	0.016	0.023
176	L C	0.892	0.015	0.111
177	I C	0.549	0.008	0.446
178	P E	0.215	0.009	0.759
179	L E	0.151	0.013	0.812
180	L E	0.052	0.005	0.889
181	G E	0.035	0.003	0.912
182	I E	0.063	0.010	0.870
183	D E	0.189	0.019	0.729
184	V H	0.449	0.526	0.051
185	W H	0.280	0.791	0.039
186	E H	0.310	0.675	0.012
187	H C	0.832	0.163	0.015
188	A H	0.440	0.547	0.007
189	Y H	0.432	0.551	0.013
190	Y H	0.172	0.815	0.009
191	L H	0.178	0.808	0.010
192	Q H	0.438	0.546	0.015
193	Y C	0.726	0.262	0.015
194	K C	0.854	0.138	0.015
195	N C	0.872	0.136	0.010
196	V C	0.925	0.091	0.003
197	R C	0.972	0.028	0.002
198	P H	0.071	0.928	0.000
199	D H	0.065	0.933	0.003
200	Y H	0.015	0.985	0.001
201	L H	0.008	0.992	0.001
202	K H	0.014	0.987	0.001

203 A H	0.024	0.977	0.002
204 I H	0.046	0.955	0.003
205 W H	0.029	0.973	0.013
206 N H	0.128	0.845	0.052
207 V C	0.496	0.382	0.055
208 I C	0.534	0.388	0.076
209 N C	0.955	0.036	0.014
210 W H	0.035	0.962	0.001
211 E H	0.022	0.978	0.001
212 N H	0.011	0.989	0.000
213 V H	0.004	0.996	0.000
214 T H	0.003	0.997	0.000
215 E H	0.003	0.997	0.000
216 R H	0.005	0.996	0.000
217 Y H	0.009	0.992	0.000
218 M H	0.011	0.990	0.000
219 A H	0.042	0.961	0.001
220 C H	0.144	0.861	0.004
221 K H	0.459	0.548	0.003
222 K C	0.999	0.000	0.000

```
# PSIPRED HFORMAT (PSIPRED V4.0)

Conf: 935777787266788646503577019999999946661030899998862888999999
Pred: CCHHHHHHHHHHHHHHHHHHHCCCCCCCCCCCCCCCCCCCCCCHHHHHHHHHHHHHHHHH
  AA: MLSRAVCGTSRQLAPVLGYLGSRQKHSLPDLPYDYGALEPHINAQIMQLHHSKHHAAYVN
              10        20        30        40        50        60

Conf: 999999999999985995799998898971565231430545148999999999389999
Pred: HHHHHHHHHHHHHHCCCHHHHHHHHHHHHHCCCCCHHCCHHCCCCCCCCCCCCCHHHHHH
  AA: NLNVTEEKYQEALAKGDVTAQIALQPALKFNGGGHINHSIFWTNLSPNGGGEPKGELLEA
              70        80        90       100       110       120

Conf: 999818999999999999975688847999998999929999769999987778971568
Pred: HHHHHCCHHHHHHHHHHHHHHCCCCCEEEEEEECCCCEEEEEECCCCCCCCCCCCCCEEE
  AA: IKRDFGSFDKFKEKLTAASVGVQGSGWGWLGFNKERGHLQIAACPNQDPLQGTTGLIPLL
             130       140       150       160       170       180

Conf: 885053611661477898899999971199999999999709
Pred: EEEHHHCHHHHHCCCCCHHHHHHHHHCCCHHHHHHHHHHHHC
  AA: GIDVWEHAYYLQYKNVRPDYLKAIWNVINWENVTERYMACKK
             190       200       210       220
```

<

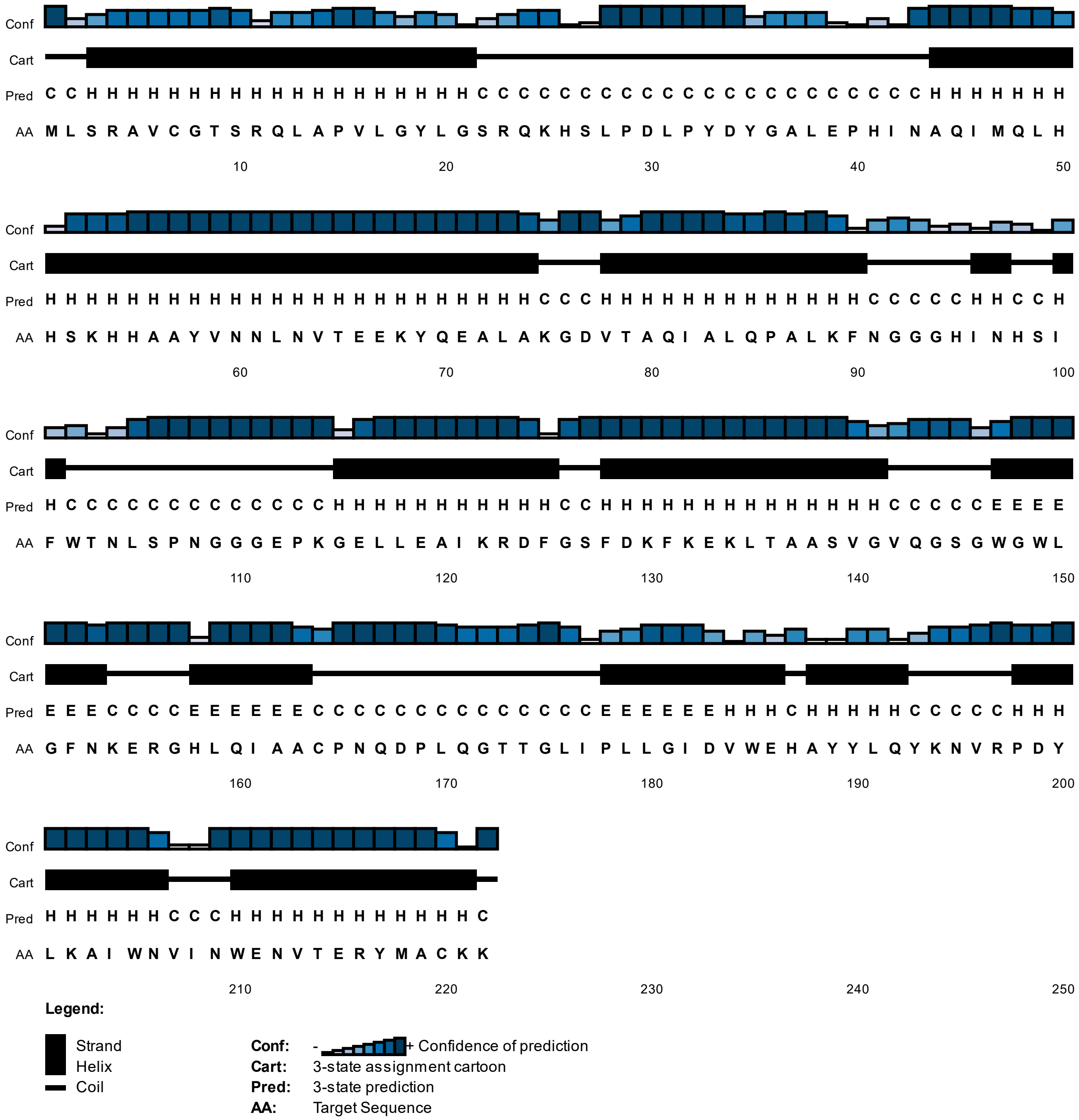

In the supplementary files we also enclose two videos:

Movie S1: MD simulation video of the Ala-MnSOD variant around the time where the Val-MnSOD exhibits a helical breakdown. The protein is presented in a green cartoon model while the MTS sequence is highlited in a blue-colored cartoon model. The Mn ion is depicted as a purple colored sphere.

Movie S2: MD simulation video of the Val-MnSOD variant around the time where it exhibits a helical breakdown. The protein is presented in a green cartoon model while the MTS sequence is highlited in an orange-colored cartoon model. The Mn ion is depicted as a purple colored sphere.
